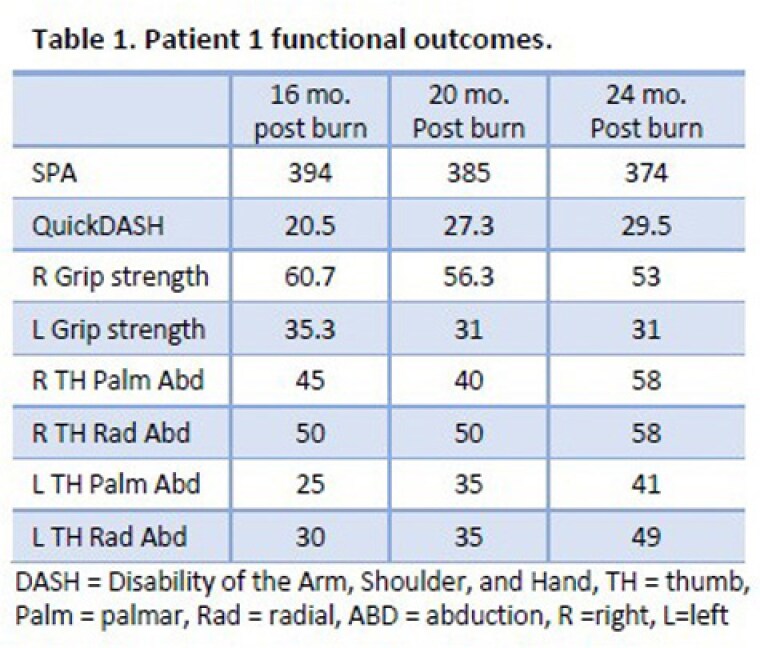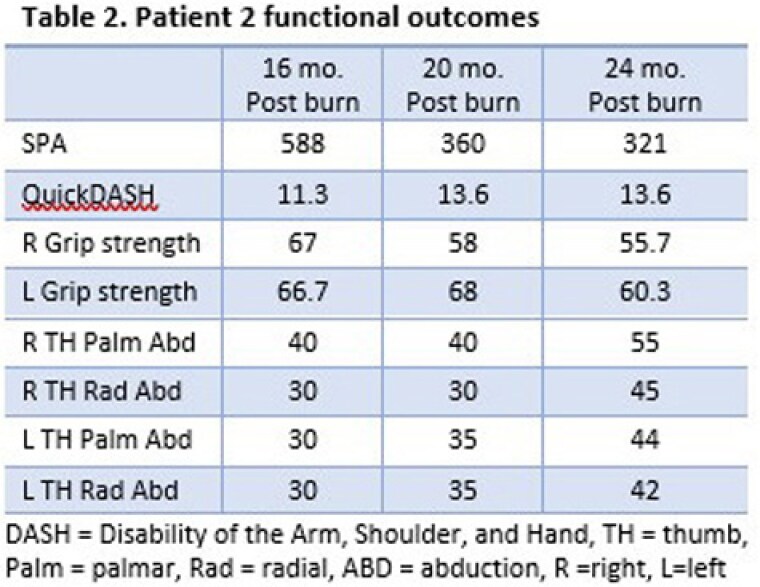# 700 Use of an Occupation-based Upper Extremity Outcome Assessment to Optimize Functional and Surgical Outcomes

**DOI:** 10.1093/jbcr/iraf019.329

**Published:** 2025-04-01

**Authors:** Jill Cancio, Leopoldo Cancio

**Affiliations:** United States Army Institute of Surgical Research Burn Center; United States Army Institute of Surgical Research Burn Center

## Abstract

**Introduction:**

Over 50% of burn survivors have hand burns, placing physical function and psychosocial wellbeing at risk. Thus, accurate assessment of hand function is critical. To date, the primary emphasis in burn rehabilitation has been the biomedical model of care. By contrast, engaging clients in occupational activities as a primary assessment method enables us to assess function in a more meaningful manner. Here, we describe a novel tool based on occupational activities, the Suitcase Packing Activity (SPA), to assess hand and upper extremity function in 2 burn survivors.

**Methods:**

Case series. The SPA is a standardized, time-based physical performance assessment that utilizes a combination of bimanual and unilateral upper extremity tasks to pack a suitcase.

**Results:**

Two men were burned in a shipboard fire and received initial care at this burn center. Patient 1 was 27 years old with 86% total body surface area (TBSA) burns; patient 2 was 29 years old with 70% TBSA burns. The SPA was completed at 16, 20, and 24 months postburn (See Table). Patient 1 sustained deeper burns to his hands and underwent 8 acute and 5 reconstructive surgeries focused on hand function; patient 2 underwent 3 acute and 3 reconstructive surgeries focused on hand function. In both patients, observation and videorecording by the therapist of SPA testing identified functional deficits in completing complex grip and pinch patterns (i.e., inability to use a lateral pinch pattern to manipulate and load a pill case, difficulty opening and manipulating a shampoo bottle). This led to reprioritization of surgical procedures by the plastic surgeon—for example, first web-space contracture releases were prioritized by both the patients and the surgeon following SPA performance.

**Conclusions:**

SPA assessment was leveraged to 1) document the impact of injury severity and location on functional changes over time; and 2) guide operative planning. Although Patient 1 has demonstrated less functional improvement than patient 2, the SPA was integral in prioritizing both functional goals and operative strategy.

**Applicability of Research to Practice:**

The SPA is valuable not only because it provides a numerical rating to quantify function but also it involves a therapist observing the individual completing functional, real-world tasks that are required in daily life. Work is underway to validate this tool in the burn population.

**Funding for the Study:**

N/A